# Multimodal postpartum imaging of a severe case of Couvelaire uterus

**DOI:** 10.1515/crpm-2021-0013

**Published:** 2022-06-07

**Authors:** Josef Jackson, Verghese George, Jennifer McKinney, Karin A. Fox

**Affiliations:** Department of Obstetrics and Gynecology, Baylor College of Medicine and Texas Children’s Hospital Pavilion for Women, Houston, TX, USA; Department of Radiology, Baylor College of Medicine and Texas Children’s Hospital Pavilion for Women, Houston, TX, USA

**Keywords:** Couvelaire uterus, magnetic resonance imaging, retained placenta, ultrasound

## Abstract

**Objectives:**

Placental abruption occurs when a normally implanted placenta prematurely separates, causing rupture of decidual spiral arteries and retroplacental hemorrhage. Estimates place the incidence of placental abruption somewhere between 0.22% and 1% of all deliveries. Clinical abruption represents a spectrum from mild to the most severe form, in which blood can extravasate into or through the myometrium, the broad ligament, or the peritoneum, causing the uterus and surrounding structures to take on a blue discoloration. This phenomenon is a clinical entity known as Couvelaire uterus, so named because it was first described by French physician Alexandre Couvelaire in the early 20th century as “uteroplacental apoplexy.” Its incidence is difficult to estimate because it has classically been diagnosed only by direct intraoperative visualization. Imaging is not usually indicated in this clinical setting, so radiologic correlation with operative findings has not been previously described.

**Case presentation:**

In this report, we discuss the case of a multipara who presented with abdominal pain and vaginal discharge several days after a classical cesarean delivery. Her prolonged and complex clinical course led to evaluation via several radiologic modalities. At first, a focal placenta accreta or retained products of conception were suspected, however these diagnoses did not correlate with the patient’s reported intraoperative findings of a clean endometrial cavity or with histopathology that was consistent with massive abruption.

**Conclusions:**

The clinical presentation and features identified on multimodal imaging were ultimately most consistent with the patient’s intraoperative diagnosis of Couvelaire uterus.

## Introduction

Placental abruption occurs when a normally implanted placenta prematurely separates, causing rupture of decidual spiral arteries and retroplacental hemorrhage [1]. Estimates place the incidence of placental abruption somewhere between 0.22% and 1% of all deliveries [1], [2], [3]. Clinical abruption represents a spectrum from mild to the most severe form, in which blood can extravasate into or through the myometrium, the broad ligament, or the peritoneum, causing the uterus and surrounding structures to take on a blue discoloration. This phenomenon is a clinical entity known as Couvelaire uterus, so named because it was first described by French physician Alexandre Couvelaire in the early 20th century as “uteroplacental apoplexy” [4]. Its incidence is difficult to estimate because it has classically been diagnosed only by direct intraoperative visualization. Imaging is not usually indicated in this clinical setting, so radiologic correlation with operative findings has not been previously described. In this report, we discuss the case of a multipara who presented with abdominal pain and vaginal discharge several days after a classical cesarean delivery. Her prolonged and complex clinical course led to evaluation via several radiologic modalities. At first, a focal placenta accreta or retained products of conception were suspected, however these diagnoses did not correlate with the patient’s reported intraoperative findings of a clean endometrial cavity or with histopathology that was consistent with massive abruption. The clinical presentation and features identified on multimodal imaging were ultimately most consistent with the patient’s intraoperative diagnosis of Couvelaire uterus. The patient whose case is described below gave written informed consent for the publication of this case report and the images contained therein. Our local institutional review board does not require board approval for case reports or case series with fewer than 3 patients.

## Case presentation

A 36-year-old woman, gravida 2 para 1, with no significant medical history except for one prior low-transverse cesarean delivery at term for arrest of dilatation, presented at a gestational age of 24 weeks 5 days for preterm premature rupture of membranes (PPROM) and chorioamnionitis. She underwent an emergent classical cesarean delivery due to non-reassuring fetal heart tones within 1 h of admission. Placental abruption and Couvelaire uterus were diagnosed intraoperatively, with the fetus and anterior placenta delivered en caul. Pathologic examination of the placenta indicated “abnormal placental separation” and ascending infection. She completed standard antibiotic therapy with ampicillin 2 g every 6 h and gentamicin 5 mg/kg every 24 h for her intrauterine infection until she was afebrile for 24 h, and had an otherwise uncomplicated postpartum course with discharge home in good condition on postoperative day 3.

**Figure 1:**
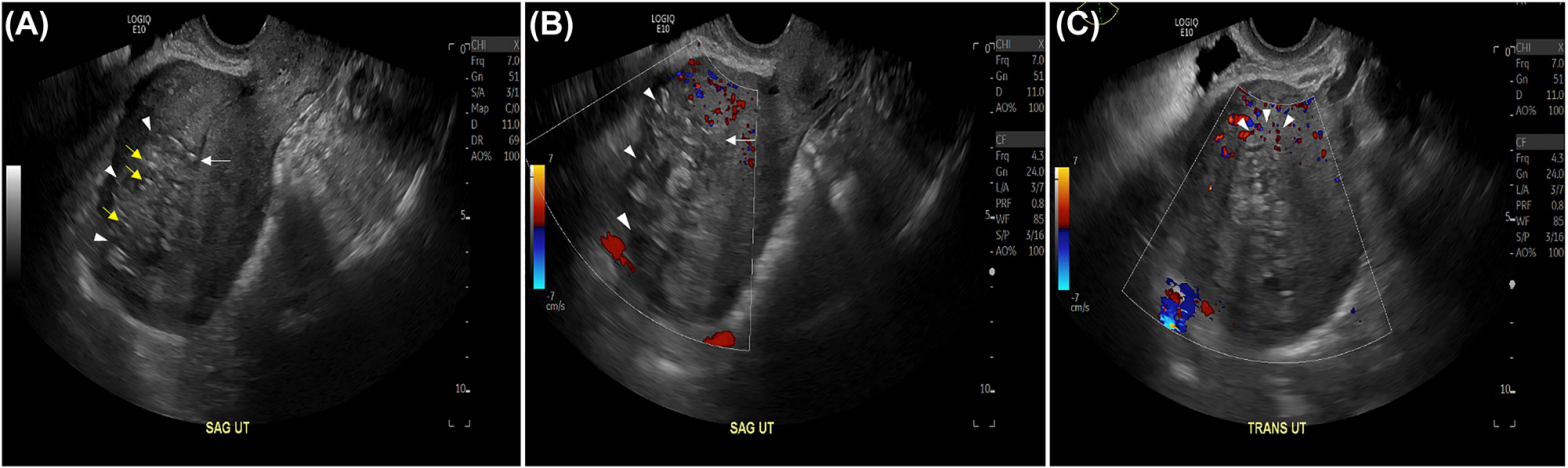
Sagittal (A, B) and axial (C) transvaginal ultrasound images obtained on post-operative day 18. The images demonstrate ill-defined infiltrating echogenicity (white arrow heads) that extends into the subserosal anterior myometrium, with minimal protrusion into the endometrial canal (white arrow), raising initial concern for potential invasive placentation. However, no vascularity is noted within the area on Doppler (B, C). Parallel linear echogenicities (yellow arrows A) are consistent with sutures indicating the hysterotomy site.

She presented again on postoperative day 16 complaining of severe lower abdominal pain and foul-smelling vaginal discharge and was evaluated by a new team of physicians. Upon presentation, her physical examination revealed a well-healed skin incision, no abdominal distention, and marked fundal tenderness. She was afebrile and normotensive and had a high-normal white blood cell count at 9.85 × 10^9^/L (normal: 4.5–11.0 × 10^9^). Her urinalysis and urine cultures were negative, and there was no other obvious source of abdominal pain. She was diagnosed with postpartum endometritis, admitted, and started on intravenous gentamycin and clindamycin. After 36 h of antibiotic therapy, her lower abdominal pain persisted and she continued to have abnormal dark vaginal discharge, so transvaginal and transabdominal ultrasound were performed to investigate for retained products of conception as a potential cause. The ultrasound demonstrated an ill-defined, infiltrating echogenicity extending from the anterior junctional zone into the subserosal myometrium that protruded into the endometrial canal proximally (Figure 1). The endometrial canal otherwise appeared unremarkable, without evidence of abnormal fluid or air. These findings were not felt to be consistent with endometrial infection, but rather with postsurgical changes. Because of the immediate clinical presentation, ultrasound findings, and the pathology report reading “abnormal placental separation”, initially there was concern for focal placenta accreta. However, the patient had not experienced significant vaginal bleeding and her placenta had delivered intact without difficulty – in fact, it had delivered en caul and the intrauterine cavity was cleared of clots and debris, and the mass seen on ultrasound was avascular, atypical for focally invasive placenta. Upon closer review of the complete pathology report, the “abnormal placental separation” appeared to describe premature placental separation rather than lack of separation, which was initially presumed. Serum quantitative beta-hCG was drawn and resulted at less than 1.5 milli-international units, below the limit of detection, and supportive of complete placental removal.

**Figure 2:**
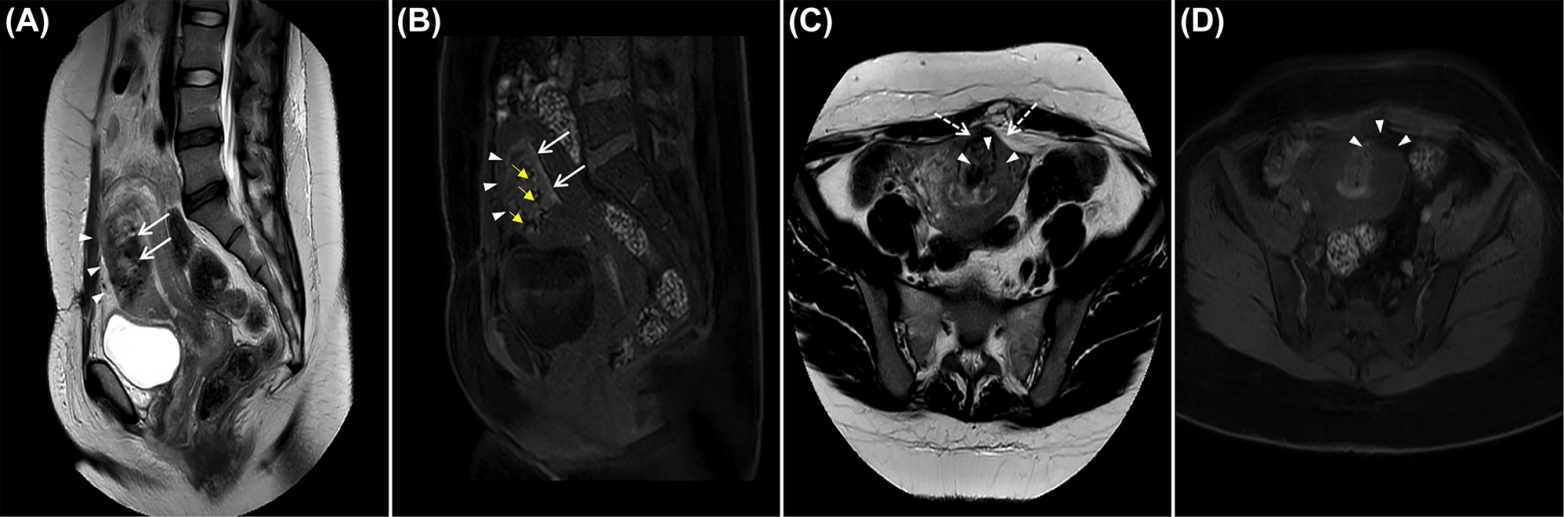
Sagittal and axial T2 MRI images (A, C) obtained on post-operative day 20. The images demonstrate a large, relatively ill-defined hypointense area involving almost the entire anterior myometrium in the midline with a sharp line of demarcation posteriorly at the anterior junctional zone (white arrows). This corresponds to sagittal and axial precontrast T1 MRI images (B, D) from the same scan that show an ill-defined infiltrating hyperintensity in the same area (white arrow heads). A dark “hemosiderin ring” is noted on T2 (white dashed arrows). Susceptibility artifacts (yellow arrows B) indicate the hysterotomy site.

**Figure 3:**
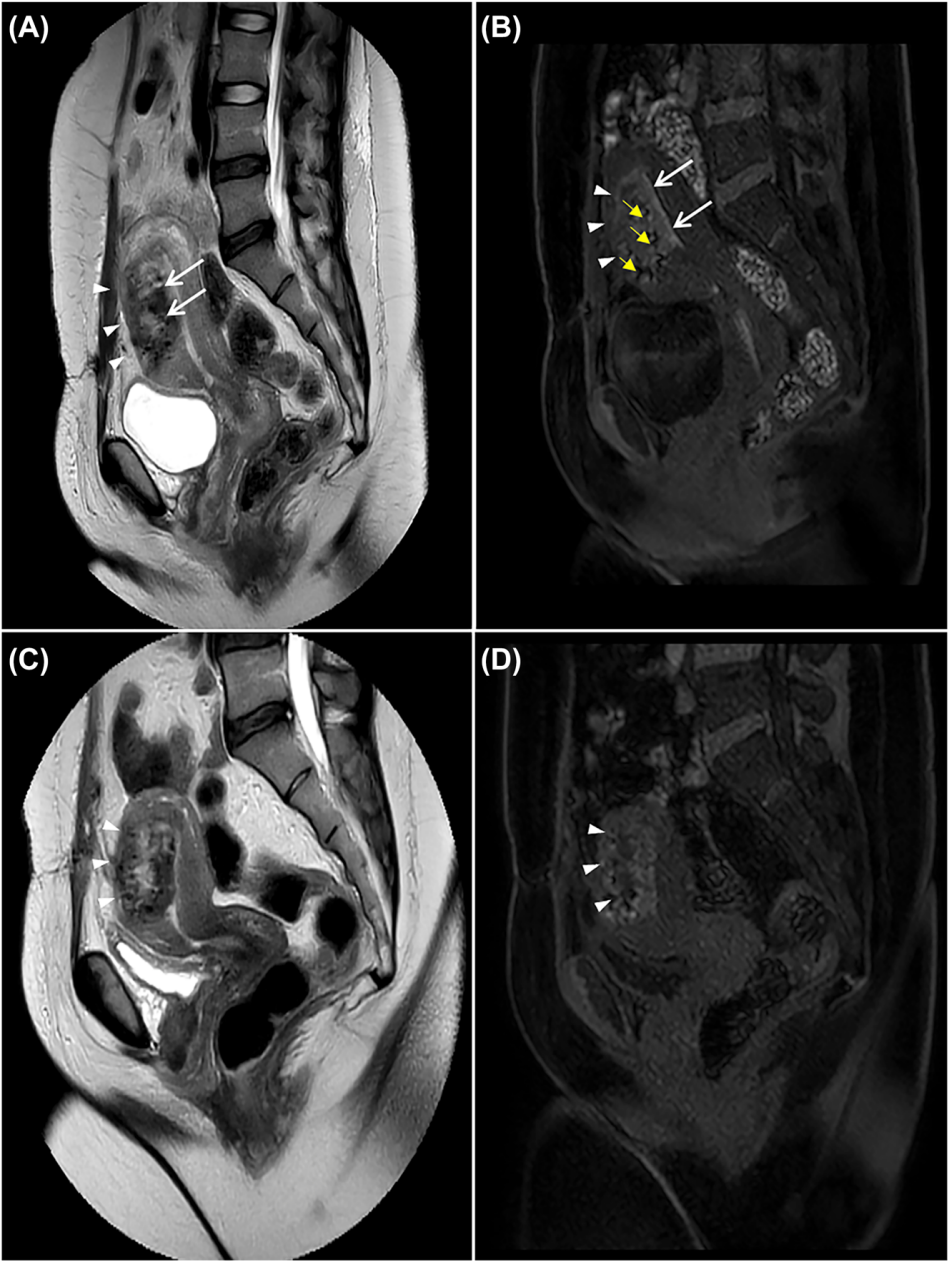
Sagittal precontrast (A) and subtracted post-contrast (B) T1-weighted images obtained on post-operative day 20. The images show completed absence of enhancement in the affected anterior myometrium. Note normal enhancement of the rest of the uterus. Due to characteristic appearances, these findings were consistent with a contained intramyometrial hematoma, related to Couvelaire uterus or post-surgical changes from classical cesarean delivery. Repeat MRI imaging two weeks after the initial study demonstrates interval evolution of the anterior intramyometrial hematoma. Sagittal (C, D) T2 and pre-contrast T1 weighted images demonstrate that the area is slightly smaller than the previous study, has increased T2 signal within its central aspect (C white arrow heads), possibly indicating interval liquefaction, and has a more prominent hemosiderin ring with reduced prominence of the precontrast T1 hyperintensity (D white arrow heads), especially along the anterior junctional zone. Findings are again consistent with diagnosis of intramural hematoma in the setting of the clinical diagnosis of Couvelaire uterus. There was no evidence of myometrial necrosis or abscess formation.

**Figure 4:**
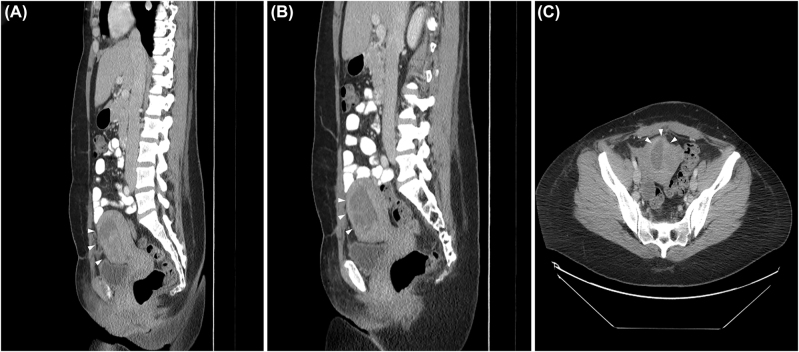
Sagittal (A, B) and axial (C) postcontrast CT images obtained on post-operative day 20. The images reveal a large heterogeneous area with patchy foci of hyperattenuation involving the anterior myometrium (white arrow heads), which corresponds to same area of abnormality on previous ultrasound and MRI imaging. These findings again support the diagnosis of large dissecting anterior intramural hematoma. The hysterotomy site is suboptimally identified on CT.

By hospital day four, pain and dark vaginal discharge persisted, despite normalization of the white blood cell count and continued absence of fever. Her prolonged clinical course with only subtle improvement with antibiotic therapy raised concern for myometrial necrosis, septic pelvic thrombophlebitis, or a non-gynecologic intraabdominal pathology such as appendicitis. Magnetic resonance imaging (MRI) with and without contrast was performed on post-operative day 20 to evaluate for these diagnoses. Therapeutic low-molecular weight heparin was initiated, and her antibiotic coverage was broadened with a plan to continue antibiotic therapy until 48 h without fever and with improvement in fundal tenderness. MRI revealed no evidence of septic pelvic thrombophlebitis but did show a large area of non-enhancement predominantly in the midline of the anterior myometrium, with a sharp posterior margin at the anterior junctional zone. This area revealed an infiltrating pattern of poorly defined T1 hyperintensity on the pre-contrast sequences that corresponded with the area of ill-defined avascular echogenicity seen on the prior ultrasound (Figures 2 and 3). Such an appearance is not typical for focal accreta which typically appears as an infiltrating mass-like process with areas of internal vascularity. Non-enhancing T1 hyperintensity involving the previously retroplacental myometrium indicates blood products in this clinical context, and imaging findings were hence considered consistent with a large, dissecting anterior intramural hematoma, likely related to Couvelaire uterus or postsurgical changes following classical cesarean delivery. Myometrial necrosis was considered due to the lack of enhancement in the affected anterior myometrium, which is an expected finding in a recent hematoma. There was no evidence of extra-serosal extension of the hematoma and no hemoperitoneum was identified. A contrast-enhanced computed tomography (CT) of the abdomen and pelvis was performed the same day because the MRI showed trace fluid around the cecum and appendix, which was concerning for an inflammatory process. On CT the appendix was unremarkable and these findings were ultimately thought to be consistent with a susceptibility artifact. The CT images also showed a large, relatively hypodense area in the anterior myometrium that corresponded with the same area of abnormality on ultrasound and MRI (Figure 4). On correlation with the intraoperative observations, the findings on multimodality imaging of a large dissecting anterior intramural hematoma were ruled most consistent with the diagnosis of Couvelaire uterus.

By hospital day six, the patient’s pain markedly improved, her vital signs and laboratory findings normalized, and she was discharged home with oral clindamycin to complete a ten-day course of antibiotics, in case of infection of the intramural hematoma. A follow-up MRI performed 2 weeks after the initial study showed interval evolution of the hematoma, without evidence of infection or abscess formation in the anterior myometrium.

## Discussion

Couvelaire uterus has been defined as the widespread extravasation of blood into the myometrium, and sometimes the broad ligament, adnexal structures, and peritoneum, as a result of placental abruption [1]. The extravasated blood may inhibit the uterus’s contractility and cause atony, and the consumption of clotting factors associated with placental abruption may also result in disseminated intravascular coagulation [5]. Because of these potential risks, it is important that clinicians recognize Couvelaire uterus and closely monitor patients for potentially life-threatening complications at and after delivery. The diagnosis has historically been made only via direct visualization of an engorged, blue-discolored uterus at the time of cesarean delivery [6], [7], [8].

Because imaging is not typically indicated in cases of suspected placental abruption, there has not been published literature that describes imaging findings in correlation with an intraoperative and histopathologic diagnosis of Couvelaire uterus. In the case presented above, ultrasound, MRI, and CT images were obtained during the evaluation and management of a complicated postoperative course. The ultrasound findings of poorly-defined vascularity at the former placental implantation site, in conjunction with the preliminary reading of the pathology report, initially raised concern for postpartum placenta accreta spectrum. This diagnosis was considered on the differential alongside other clinical entities including deep myometrial infection and septic pelvic thrombophlebitis. However, when correlated with the patient’s intraoperative course, clinical presentation, and laboratory studies, imaging findings indicative of blood products infiltrating the previously retroplacental myometrium were felt to be most consistent with the patient’s intraoperative diagnosis of Couvelaire uterus. While routine ante- and postpartum imaging is not indicated in most cases of placental abruption, the imaging findings discussed above may call for the inclusion of Couvelaire uterus in the differential diagnosis when radiologic studies are performed for other clinical indications.
